# Improving quality of life in rare diseases using disease-specific, multidisciplinary online interventions on the example of rare X-linked adrenoleukodystrophy: a randomized-controlled trial

**DOI:** 10.1177/17562864251376109

**Published:** 2025-09-28

**Authors:** Lisa Schäfer, Astrid Unterlauft, Brit Froebrich-Andreß, Carolin Wollny, Marie Rößler, Ronja Fischer, Carla Bähr, Julia Lier, Daniel T. Wasmus, Christa-Caroline Bergner, Wolfgang Köhler

**Affiliations:** Department of Neurology, Leipzig University Medical Center, Leukodystrophy Outpatient Clinic, Liebigstrasse 20a, Leipzig 04103, Germany; Department of Neurology, Leipzig University Medical Center, Leukodystrophy Outpatient Clinic, Leipzig, Germany; Department of Neurology, Leipzig University Medical Center, Leukodystrophy Outpatient Clinic, Leipzig, Germany; Department of Neurology, Leipzig University Medical Center, Leukodystrophy Outpatient Clinic, Leipzig, Germany; Department of Neurology, Leipzig University Medical Center, Leukodystrophy Outpatient Clinic, Leipzig, Germany; Department of Neurology, Leipzig University Medical Center, Leukodystrophy Outpatient Clinic, Leipzig, Germany; Department of Neurology, Leipzig University Medical Center, Leukodystrophy Outpatient Clinic, Leipzig, Germany; Department of Neurology, Leipzig University Medical Center, Leukodystrophy Outpatient Clinic, Leipzig, Germany; Department of Neurology, Leipzig University Medical Center, Leukodystrophy Outpatient Clinic, Leipzig, Germany; Department of Neurology, Leipzig University Medical Center, Leukodystrophy Outpatient Clinic, Leipzig, Germany; Department of Neurology, Leipzig University Medical Center, Leukodystrophy Outpatient Clinic, Leipzig, Germany

**Keywords:** adrenoleukodystrophy (X-ALD), multidisciplinary online intervention, myeloneuropathy, quality of life (QoL), rare diseases (RDs), symptomatic women

## Abstract

**Background:**

People with rare diseases (RDs) often require intensive multidisciplinary care in disease-specific centers of excellence (CoE). However, access is limited for most patients living remotely. X-linked adrenoleukodystrophy (X-ALD) is a genetic RD leading to demyelination of the central and peripheral nervous system.

**Objectives:**

This randomized-controlled trial tested the feasibility, acceptance, and effectiveness of a multidisciplinary online intervention provided by a CoE on the quality of life (QoL) and well-being of symptomatic women with X-ALD.

**Design:**

Single-center, randomized-controlled clinical trial involving 68 German-speaking women with symptomatic X-ALD.

**Methods:**

Participants were randomized into an experimental group (EG, *n* = 34) receiving 12-month online intervention SMART-ALD and a waiting-list control group (WL-CG, *n* = 34) receiving 6-month SMART-ALD after a 6-month waiting period. Within SMART-ALD, participants were offered regular web-based neurological, social, psychological, and nutritional counseling and fitness training provided by the Leukodystrophy Outpatient Clinic at Leipzig, Germany. Group, time, and interaction effects on primary (self-reported QoL) and secondary (physical and mental health) outcomes after 6-month SMART-ALD were tested by repeated measures ANOVAs.

**Results:**

One WL-CG participant dropped out after the waiting period and was excluded from the final analysis. Significant QoL improvements in the EG versus WL-CG were found on self-reported mental health (mean difference (MD): 5.4, 95% confidence interval (CI) (2.8, 13.6), *p* = 0.020, η^2^ = 0.08) and vitality (MD: 8.8, 95% CI (0.1, 17.4), *p* = 0.002, η^2^ = 0.14). Further significant interaction effects emerged for improved knowledge about nutrition (MD: 0.4, 95% CI (−0.7, 1.4), *p* = 0.002, η^2^ = 0.15), socio-medical benefits (MD: 1.8, 95% CI (0.5, 3.0), *p* = 0.033, η^2^ = 0.07), and intense physical activity (MD: 2.2, 95% CI (−3.9, 8.4), *p* = 0.024, η^2^ = 0.10).

**Conclusion:**

The study shows that easily accessible, multidisciplinary online interventions provided by the CoE have the potential to improve the QoL in people with RDs by providing regular access to specialized care.

**Trial registration:**

This study was registered on ClinicalTrials.gov (https://clinicaltrials.gov/study/NCT04687007).

## Plain language summary

Improving the quality of life of people with rare diseases through the use of multi-professional and easily accessible online care programs

People with rare diseases often require disease-specific and multi-professional care, but regular access is limited for those who live far from their expert centers. One rare disease is X-linked adrenoleukodystrophy (X-ALD), a genetic neurological disorder that affects the nerves in the brain and body. The aim of this study was to investigate whether an online lifestyle program can help women with symptoms of X-ALD to improve their quality of life and general well-being. The study involved 68 German-speaking women with symptomatic X-ALD. They were divided into two groups: One group received an online lifestyle program called SMART-ALD for one year, while the other group waited for six months before starting the same program. The SMART-ALD program offered neurological counseling, socio-medical counseling, psychological support, nutritional counseling, and individualized fitness training. Patients could choose which counseling sessions they wanted to attend depending on their current life situation and needs. All sessions were conducted online by X-ALD experts from the Leukodystrophy Outpatient Clinic in Leipzig, Germany. After six months, the results showed that the women who had participated in the online program reported significant improvements in their mental health and vitality compared to those who had not yet participated. They also had better knowledge of nutrition and social benefits and were more physically active. In summary, this study shows that online health interventions offered by expert centers can significantly improve the quality of life of people with rare diseases such as X-ALD. This approach can help overcome the barriers to accessing specialized care, making such interventions potentially valuable for many patients with rare neurological diseases.

## Introduction

Around 446 million people worldwide live with rare diseases (RDs),^1^ which usually have a chronic and complex clinical picture requiring regular, multidisciplinary treatment in a specialized center of excellence (CoE).^2^ However, regular access to these CoE is limited for patients living remotely, and knowledge about diagnosis and treatment standards is often insufficient among those providing regular care.^3^ X-linked adrenoleukodystrophy (X-ALD) is a RD (estimated birth incidence: 1:15,000) caused by mutations in the *ABCD1* gene that leads to a pathological accumulation of very long chain fatty acids (VLCFA) in all body fluids and tissues, causing degeneration of spinal cord tracts, peripheral nerve damage, dysfunction of adrenal cortex, and inflammation of central nervous system (CNS) white matter.^4^ The most common phenotype of X-ALD in adulthood is adrenomyeloneuropathy (AMN), clinically characterized by sensory ataxia and spastic paraparesis causing progressive balance and gait disorders, and bladder, sexual, and bowel dysfunction. Adrenal insufficiency occurs mainly in male (up to 80%) versus female (<1%) patients.^4,5^ Sex effects in X-ALD are also seen in the CNS involvement: The risk of developing cerebral X-ALD (CALD), that is, rapidly progressive inflammatory destruction of the white matter, in males is estimated 35% during childhood and up to 60% over the entire live span, whereas this is very rare in women (<1%).^4,5^

Additionally, women with X-ALD experience a later onset of myeloneuropathy and milder symptoms as men, which may explain why they have long been regarded as mere gene carriers without significant disease burden. However, recent clinical studies have identified myeloneuropathy in up to 80% of women after the age of 40, limiting their quality of life (QoL) and increasing the prevalence of physical and psychological comorbidities.^6–8^ Furthermore, women caring for sons with CALD reported diminished mental health, reduced opportunities for social and professional participation, and financial burdens.^7,9^ These findings suggest that the clinical care of symptomatic women requires a multidisciplinary approach in which psychosocial needs for specific patient groups are addressed.^8,10^ Female symptomatic X-ALD gene carriers still experience a particularly high rate of mis- or undertreatment outside CoE.^8,11^ This group could significantly benefit from web-based clinical programs to ensure specialized care is accessible with sufficient frequency, regardless of mobility restrictions, social and psychological limitations, or lack of transportation options.

This randomized-controlled study aimed to test the feasibility, acceptance, and effectiveness of a disease-specific, multidisciplinary, easily accessible online lifestyle intervention (SMART-ALD) on the QoL and well-being of symptomatic women with X-ALD. We hypothesized that participants report increased QoL after 6 months of receiving SMART-ALD compared to a control group treated as usual. Secondary hypotheses were that 6-month SMART-ALD participation has a positive effect on self-reported physical and mental health outcomes, patients’ perceived self-efficacy, and objectively measured levels of daily physical activity. In addition, we investigated whether 12-month SMART-ALD participation maintains intervention effects and explored how participation frequency and person-related characteristics influence the effects of this intervention.

## Materials and methods

### Participants

Recruitment of eligible participants took place between May and October 2022 and included web-based outreach via the web platform *Leuconnect* run by the patient organization ELA International (www.leuconnect.com), and local advertising at the Leukodystrophy Outpatient Clinic. Inclusion criteria were proven X-ALD (elevated VLCFA values or mutation in *ABCD1* gene), female sex, the presence of myeloneuropathy (Adult ALD Clinical Score (AACS) ⩾2),^12^ age ⩾18 years, German language skills, and meeting the technical requirements to conduct regular video consultations using the open source web conferencing system BigBlueButton™ from BigBlueButton Inc. Exclusion criteria were any medical condition that may interfere with the study (e.g., acute psychosis; current pregnancy; severe liver, kidney, active infections, or major heart diseases that have an estimated life expectancy of <6 months).

**Figure 1.**
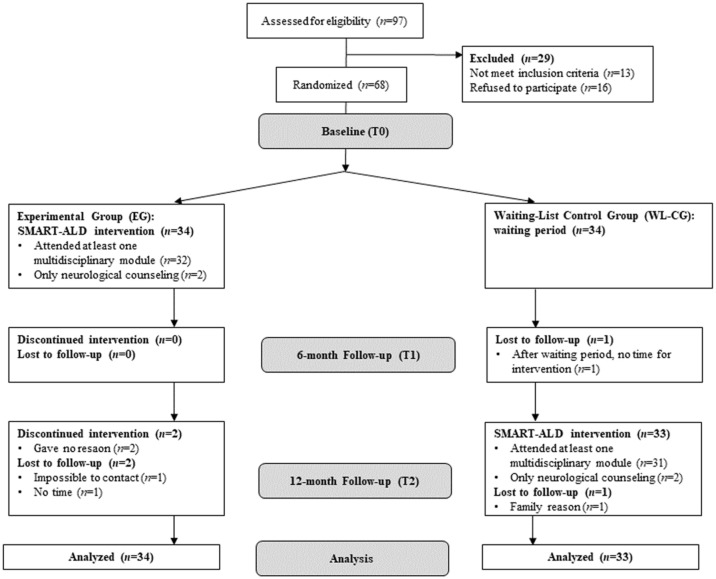
CONSORT flowchart of participants. CONSORT, Consolidated Standards of Reporting Trials.

A priori sample size calculation revealed that, given small-to-medium sized effects (*f* = 0.20) for changes in self-reported QoL (SF-36) from pre- to 6-month-post-SMART-ALD, a total sample size of *n* = 60 participants was required for detecting within-between interactions in repeated measures analyses of variance (rmANOVAs) with adequate power of 85%. Assuming data loss due to drop-out and invalid data in up to 15%, study enrollment was set to *n* = 34 individuals per group. Detailed participant flow is depicted in the Consolidated Standards of Reporting Trials diagram (Figure 1).

### Study design

**Figure 2.**
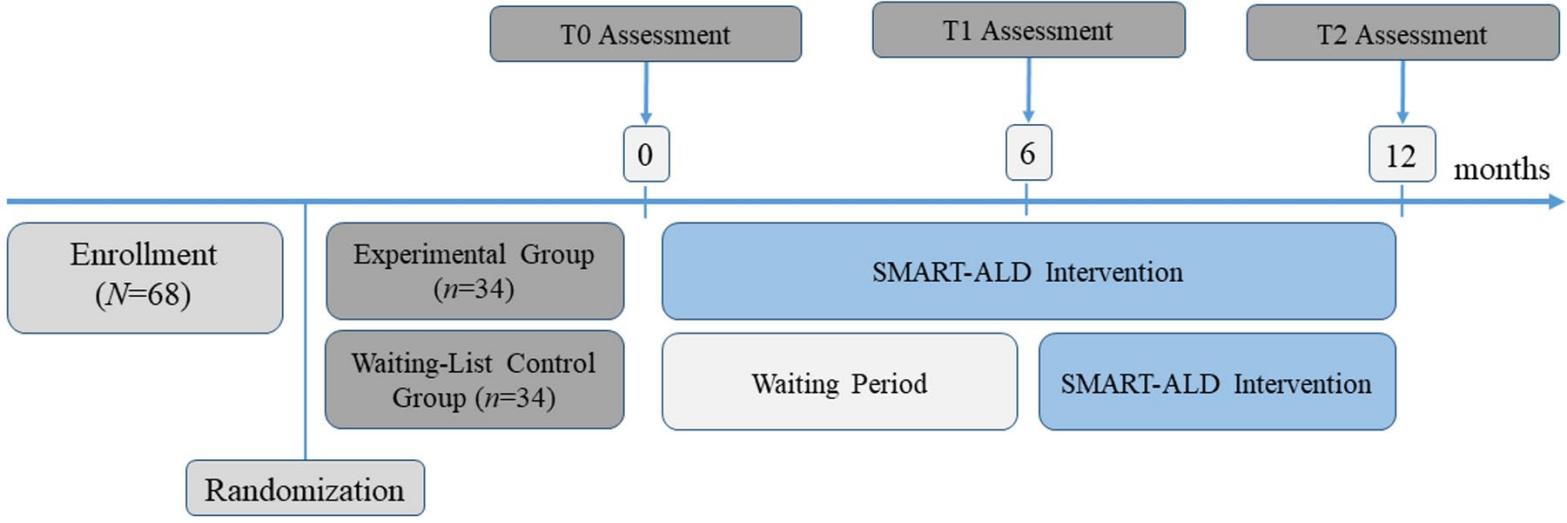
Graphic representation of the study design.

This monocentric, randomized-controlled clinical trial used a within–between subject design with participants being randomly assigned at the beginning of the study in a 1:1 ratio to either an experimental group (EG) or a waiting-list control group (WL-CG; between-subjects), each measured at three different time points (within-subjects; Figure 2). Randomization was conducted at pretreatment by an independent researcher using an online randomization tool (http://www.randomization.com). The EG received 12 months of SMART-ALD, while the WL-CG had to wait 6 months before also receiving 6 months of SMART-ALD. This study was registered on ClinicalTrials.gov (https://clinicaltrials.gov/study/NCT04687007).

### Diagnostic assessments

All participants were invited to three web-based diagnostic assessments: T0 (baseline), T1, and T2. The following assessments were conducted at each time point.

#### Neurological exploration

Participants received a diagnostic video call by a trained neurologist with X-ALD expertise, in which individuals’ medical history, neurological status, and current symptomatic treatment were assessed. Myeloneuropathy was determined by an interview-based AACS (range: 0–12),^12^ which assesses deficits in motor, bladder, and sensory functions. Cerebral domain was omitted due to the low risk of CALD in women.^4,6^ Furthermore, patients’ current socio-medical support (e.g., appropriateness of care level, provision of state aids) was reviewed.

#### Self-report questionnaires

Participants completed self-report questionnaires on the web platform *Leuconnect*. Health-related QoL was measured using the total scores of the physical and mental components of the Short Form (36) Health Survey (SF-36).^13^ Health component scores were assigned to standardized *T* values (norm range: 40–60) according to age- and sex-matched norms. Additionally, the total scores of the eight subscales (physical functioning, role limitations due to physical problems, bodily pain, general health, vitality, social functioning, role limitations due to emotional problems, mental health) were calculated, assessing QoL on a scale from 0.0 = lowest QoL to 100.0 = highest QoL. Bladder dysfunction was evaluated by the total score (0–48) of the International Consultation on Incontinence Questionnaire Female Lower Urinary Tract Symptoms Module (ICIQ-FLUTS).^14^ Sleeping disorders were rated by the total score (0–21) of the Pittsburgh Sleep Quality Index (PSQI).^15^ The total score (0–84) of the Modified Fatigue Impact Scale (MFIS)^16^ was used to measure the impact of fatigue on participants’ QoL. Symptoms of depression were rated by the total score (0–63) of the Beck Depression Inventory (BDI-II).^17^ Participants’ self-efficacy, defined as self-beliefs of coping with demanding situations due to personal competence, was assessed by the total score (10–40) of the General Self-Efficacy Scale (GSE).^18^ Satisfaction with the current disease-specific treatment of physical and psychological symptoms and self-rated knowledge about nutritional recommendations were recorded on a scale of 1–10, with higher values indicating greater satisfaction/knowledge. In addition, participants rated their knowledge of socio-medical disability benefits (e.g., reduced earning capacity, financial assistance and compensation for disadvantages, long-term care insurance benefits, social participation benefits) and their confidence in applying for these aids from authorities and health insurance companies on a scale from 1 = not at all informed/confident to 10 = very well informed/confident using five items. The five items were combined into a total mean score, which indicates the participants’ knowledge of socio-medical disability benefits.

#### Fitness tracker data

To objectively measure patients’ physical activity, all participants were provided with free fitness trackers (Fitbit Inspire 2™ from Fitbit Inc.) at the beginning of the study and asked to wear them continuously for 4 weeks at each assessment point. Data collection included the average number of steps, minutes of light, moderate, and intense physical activity, as well as active calories per day over the wearing period at T0, T1, and T2, respectively.

#### Evaluation rating

At T2, all patients were asked to rate the SMART-ALD intervention in terms of their feasibility, benefit, and effort using seven items with a 5-point Likert scale. A total score was calculated with higher values indicating greater satisfaction with the lifestyle intervention.

### Intervention SMART-ALD

At the beginning of SMART-ALD (for EG: at T0, for WL-CG: at T1), an individualized treatment plan was developed based on the multidisciplinary assessment and the patient’s current life situation, needs, and wishes. This included medical treatment recommendations and an agreement on how many and which additional, non-medical SMART-ALD intervention modules the patient wanted to participate alongside. The following intervention modules were offered, with all appointments taking place online and the scope and frequency of participation being chosen by the patient and continuously adapted to changing needs over time.

#### Medical counseling

To optimize the treatment of neurological symptoms, a neurologist experienced in X-ALD made treatment recommendations and reviewed their implementation after 6 months. Moreover, patients and their local physicians had the opportunity to contact the study team directly at any time with questions.

#### Psychological counseling

Patients had the opportunity to take advantage of individual psychological counseling sessions. These could deal with specific topics (e.g., psychoeducation, coping with illness, grief, stress management, strengthening one’s own resources) but could also be used by patients as a protected space for emotional relief and reflection.

#### Social counseling

A social worker assessed the participant’s current socio-medical care situation in various areas (e.g., current housing situation, employment and disability, financial situation, social participation, care of affected family members). In the event of gaps in care, the patient was informed about state aid and, if required, actively assisted with the application process.

#### Nutritional counseling

Nutritional advice included recommendations aimed at a balanced, healthy diet. In addition, information was provided on the VLCFA content of foods, along with regular recipe suggestions sent by email. Patients had the opportunity to have individual consultations with a nutritionist who specializes in X-ALD diets. The individual consultations were based on the evaluation of a multi-day nutrition log previously kept by the patient.

#### Fitness training

The patients were offered weekly fitness training sessions in an individual or group setting. The exercises were aimed at improving the patients’ endurance, coordination, strength, and balance, as well as increasing their motivation to exercise and their self-efficacy. The intensity of the exercises was individually adapted to the patient’s fitness level and increased as the training progressed. Additionally, the exercises were made available to the patients as YouTube videos^19^ to provide them with long-term guidance on disease-specific exercises that they can integrate into their everyday lives after the study end.

### Primary and secondary outcomes

The primary outcome was defined as the change in self-reported QoL assessed by SF-36 after the 6-month SMART-ALD intervention (T1) in the EG compared to the WL-CG. Secondary outcomes included all other self-reported (questionnaires) and objective (AACS, fitness tracker) measures of improvements in physical and mental health after 6 months (controlled effects due to WL-CG) and 12 months (uncontrolled effects) of SMART-ALD intervention.

### Statistical analysis

Independent *t* tests were performed to test differences between EG versus WL-CG at baseline (T0). Controlled effects on primary and secondary outcomes were determined by rmANOVAs with the factors Group (EG, WL-CG; between-subjects) × Time (T0, T1; within-subjects). Bonferroni adjustment for multiple testing was not applied because (1) *p* values are not considered a main result and are worthless without considering the sign and size of the effects, (2) the hypotheses tested are not independent of each other and their interdependencies cannot be adequately integrated into a procedure for adjusting for multiple testing, (3) few people understand the different underlying statistical procedures for correcting for multiple testing and are aware of the significant impact on statistical power, making the results more difficult to understand. Long-term effects of SMART-ALD during 12-month participation on outcome measures within the EG were analyzed by rmANOVAs with time (T0, T1, T2) as within-subjects factor. Post hoc tests with Bonferroni correction were performed to examine pairwise differences when omnibus tests were significant. To examine the effects of participation frequency on outcome measures, first, all participants who had selected at least one multidisciplinary module at T0 (EG, *n* = 32) and T1 (WL-CG, *n* = 31) were combined into a 6-month intervention group (6m IG). Second, the variables PreSMART-ALD (EG: T0, WL-CG: T1 data) and 6m-PostSMART-ALD (EG: T1, WL-CG: T2 data) were created for each outcome measure. Third, the changes within the outcome variables after the 6-month intervention were determined as difference scores by subtracting 6m-PostSMART-ALD from PreSMART-ALD. In a final step, the 6m IG was divided into subgroups according to the number of modules taken and compared regarding Pre-SMART-ALD characteristics, difference scores, and participants’ total score of evaluation of SMART-ALD at T2 using ANOVA with post hoc testing and Bonferroni correction if the omnibus tests were significant. Pearson correlation analyses were performed to detect associations between person-related baseline characteristics (age, AACS, Pre-SMART-ALD variables), participation frequency (total time of participation in all selected modules in minutes), and participants’ evaluation of SMART-ALD on various aspects at T2.

Data were analyzed using IBM SPSS Statistics Version 29.0. All tests were two-tailed, and the significance level was set at α = 0.050. Effect sizes were estimated for all analyses and interpreted according to Cohen.^20^ The study protocol and statistical analysis plan are available as Supplemental Material (eSPA).

## Results

### Sample description

A total of *N* = 68 symptomatic Caucasian women with X-ALD (*n* = 65 German, *n* = 2 Austrian, *n* = 1 Swiss) were included. One participant in the WL-CG ended study participation after the waiting period (before T1) due to time constraints and was excluded from the final data analysis. Further, two participants in the EG did not submit complete self-report questionnaires at T1. For these two and one other participant in the EG and one participant in the WL-CG, the data from the diagnostic assessment (neurological explorations and/or self-report questionnaires) were missing at T2. At T0, both groups did not differ significantly in terms of age in years (*M*_EG_ = 55.3 ± 6.8 vs *M*_WL-CG_ = 57.0 ± 12.7; *t*(1, 48.7) = −0.670, *p* = 0.506, *d* = −0.17), education measured in school years (*M*_EG_ = 10.9 ± 1.5 vs *M*_WL-CG_ = 10.9 ± 1.5; *t*(1, 65) = −0.236, *p* = 0.814, *d* = −0.06), disease severity assessed by AACS (*M*_EG_ = 5.5 ± 2.8 vs *M*_WL-CG_ = 5.2 ± 2.5; *t*(1, 64) = 0.480, *p* = 0.633, *d* = 0.12), and number of patients also being caregivers (*n*_EG_ = 11 vs *n*_WL-CG_ = 8; χ^2^(1, *N* = 67) = 0.542, *p* = 0.462, *V* = 0.09).

### Controlled effects of 6-month SMART-ALD intervention

Detailed analysis results of all outcome variables are displayed in Table 1. Significant Group × Time effects in outcome measures were summarized graphically in Figure S1 (Supplemental Material).

**Table 1.** Outcome measures as a function of group (EG vs WL-CG) and time (T0 vs T1).

Outcome measures	EG (*n* = 34)		WL-CG (*n* = 33)		*F*	*df*	*p*	η^2^
	T0	T1	T0	T1				
	*M* (SD)		*M* (SD)					
Primary outcome								
Quality of life (SF-36)								
Physical health component	36.4 (9.6)	35.8 (9.9)	35.2 (13.0)	34.4 (11.5)	0.023	1, 64	0.880	0.00
Mental health component	45.2 (10.0)	50.6 (10.4)	46.4 (12.0)	48.5 (9.9)	2.269	1, 64	0.137	0.03
Physical functioning	47.5 (26.9)	43.6 (29.9)	46.5 (32.7)	42.4 (31.7)	0.005	1, 65	0.944	0.00
Role-physical	42.4 (38.3)	45.5 (35.1)	41.7 (43.2)	46.2 (45.1)	0.024	1, 64	0.878	0.00
Bodily pain	56.5 (23.3)	58.5 (23.0)	57.4 (27.5)	55.6 (28.8)	0.592	1, 65	0.444	0.01
General health	46.7 (19.1)	54.9 (19.6)	45.7 (17.0)	48.3 (19.0)	2.514	1, 65	0.118	0.04
Vitality	41.2 (13.3)	50.6 (18.3)	41.7 (16.4)	41.8 (17.1)	10.901	1, 65	**0.002**	0.14
Social functioning	63.6 (26.2)	70.2 (25.6)	62.9 (23.9)	65.5 (28.8)	0.794	1, 65	0.376	0.01
Role-emotional	57.6 (39.3)	68.7 (40.8)	63.6 (42.0)	73.7 (42.3)	0.007	1, 64	0.933	0.00
Mental health	63.5 (15.0)	70.2 (16.1)	64.5 (18.6)	64.7 (17.3)	5.721	1, 64	**0.020**	0.08
Secondary outcome								
Self-report questionnaires								
Bladder dysfunction (ICIQ-FLUTS)	17.6 (8.0)	17.1 (6.1)	13.6 (7.7)	14.5 (6.8)	1.343	1, 56	0.251	0.02
Sleep quality (PSQI)	7.6 (2.7)	6.8 (3.1)	8.9 (4.0)	8.4 (3.3)	0.083	1, 63	0.774	0.00
Fatigue (MFIS)	34.4 (14.0)	29.8 (15.8)	32.0 (14.7)	31.4 (16.9)	3.134	1, 63	0.082	0.05
Depression (BDI-II)	10.7 (5.7)	8.3 (6.3)	12.0 (9.5)	10.9 (8.7)	0.763	1, 63	0.386	0.01
Self-efficacy (GSE)	28.4 (5.0)	30.6 (4.5)	28.9 (5.8)	29.6 (5.3)	3.065	1, 63	0.085	0.05
Satisfaction with current treatment of physicalsymptoms (1*–10)	4.5 (2.5)	5.9 (2.6)	4.8 (2.5)	5.1 (2.4)	2.814	1, 64	0.098	0.04
Satisfaction with current treatment of psychological symptoms (1*–10)	5.4 (3.0)	6.8 (3.1)	5.3 (3.0)	5.6 (2.5)	2.804	1, 65	0.099	0.04
Knowledge of balanced nutrition (1*–10)	5.3 (2.4)	6.6 (2.0)	6.6 (2.3)	6.2 (2.3)	10.962	1, 64	**0.002**	0.15
Knowledge of socio-medical benefits for disability (1*–10)	4.9 (2.8)	6.8 (2.3)	4.3 (2.8)	5.0 (2.7)	4.769	1, 60	**0.033**	0.07
Objective measures								
Disease severity (AACS)	5.5 (2.8)	5.4 (2.5)	5.2 (2.5)	5.3 (2.3)	0.820	1, 64	0.368	0.01
Fitness tracker data								
Number of steps	8930.8 (5168.6)	9195.5 (5485.9)	7933.8 (3534.4)	7404.0 (4035.5)	1.941	1, 48	0.170	0.04
Light physical activity	299.9 (110.3)	288.5 (104.0)	286.0 (87.1)	269.4 (94.4)	0.096	1, 47	0.758	0.00
Moderate physical activity	18.4 (18.4)	17.2 (19.0)	15.2 (13.8)	9.9 (11.1)	1.996	1, 47	0.164	0.04
Intense physical activity	7.0 (7.8)	12.3 (16.7)	8.0 (10.9)	6.8 (9.8)	5.443	1, 47	**0.024**	0.10
Active calories	991.7 (455.4)	985.3 (448.1)	957.7 (338.2)	869.4 (364.4)	2.258	1, 47	0.140	0.05

Statistically significant p-values are indicated in bold. Effect size η^2^ was interpreted according to Cohen (small effect: 0.01 ⩽ η^2^ < 0.06, medium: 0.06 ⩽ η^2^ < 0.14, large: η^2^ ⩾ 0.14).^20^

AACS, Adult Adrenoleukodystrophy Clinical Score (0–12*; less favorable scores are asterisked); BDI-II, Beck Depression Inventory (0–63*); EG, experimental group; GSE, General Self-Efficacy Scale (10*–40); ICIQ-FLUTS, International Consultation on Incontinence Questionnaire Female Lower Urinary Tract Symptoms Module (0–48*; this questionnaire was only completed by patients with bladder dysfunction); MFIS, Modified Fatigue Impact Scale (0–84*); PSQI, Pittsburgh Sleep Quality Index (0–21*); SF-36, Short Form (36) Health Survey (health components: *T* values according to age- and sex-based norms, standard range: 40 ⩽ *T* ⩽ 60; subscales: 0*–100); WL-CG, waiting-list control group.

#### Primary outcome

RmANOVAs revealed a medium-to-large-sized Group × Time effect on the SF-36 subscales vitality (*p* = 0.002, η^2^ = 0.14) and mental health (*p* = 0.020, η^2^ = 0.08), with the EG versus WL-CG showing significant increases after 6-month SMART-ALD participation (mean group difference scores at T1—vitality: 8.8, 95% confidence interval (CI) (0.1, 17.4), mental health: 5.4, 95% CI (2.8, 13.6)). Significant time effects were found on the SF-36 mental health component, *F*(1, 64) = 11.229, *p* = 0.001, η^2^ = 0.15, and on the subscales physical functioning, *F*(1, 65) = 6.617, *p* = 0.012, η^2^ = 0.09, general health, *F*(1, 65) = 9.230, *p* = 0.003, η^2^ = 0.12, and social functioning, *F*(1, 65) = 4.338, *p* = 0.041, η^2^ = 0.06. Both groups reported higher values in these aspects after 6 months; however, increases in mean scores over time were only statistically significant in the EG for the mental health component (*p* = 0.001, *d* = 0.62), and the subscales general health (*p* = 0.005, *d* = 0.52) and social functioning (*p* = 0.037, *d* = 0.37). No further significant effects of group, time, or Group × Time were detected.

#### Secondary outcome

Medium-to-large-sized Group × Time effects revealed on self-reported knowledge of balanced nutrition (*p* = 0.002, η^2^ = 0.15) and socio-medical benefits for disability (*p* = 0.033, η^2^ = 0.07), and intense physical activity measured by fitness trackers (*p* = 0.024, η^2^ = 0.10), with the EG versus WL-CG showing significant increases in these aspect from T0 to T1 (mean group difference scores at T1—nutrition: 0.4, 95% CI (−0.7, 1.4), socio-medical knowledge: 1.8, 95% CI (0.5, 3.0), intense activity: 2.2, 95% CI (−3.9, 8.4)). Significant time effects were found on MFIS, *F*(1, 63) = 5.538, *p* = 0.022, η^2^ = 0.08, BDI-II, *F*(1, 63) = 6.177, *p* = 0.016, η^2^ = 0.09, GSE, *F*(1, 63) = 11.479, *p* = 0.001, η^2^ = 0.15, satisfaction with current treatment of physical symptoms, *F*(1, 64) = 6.923, *p* = 0.011, η^2^ = 0.10, and psychological symptoms, *F*(1, 65) = 6.707, *p* = 0.012, η^2^ = 0.09, and moderate physical activity per day, *F*(1, 47) = 5.024, *p* = 0.030, η^2^ = 0.10. Both groups showed improvements in these aspects over time except for moderate physical activity. Changes in mean scores from T0 to T1 were only statistically significant in the EG (MFIS: *p* = 0.005, *d* = 0.52; BDI-II: *p* = 0.022, *d* = 0.47; GSE: *p* < 0.001, *d* = −0.66; satisfaction with current treatment of physical symptoms: *p* = 0.003, *d* = −0.53; satisfaction with current treatment of psychological symptoms: *p* = 0.003, *d* = −0.72), and the decrease in moderate physical activity from T0 to T1 only in the WL-CG (*p* = 0.014, *d* = 0.44). No further significant effects of group, time, or Group × Time were detected.

### Changes within the 12-month SMART-ALD intervention

Table 2 depicts the changes in the EG in outcome measures during the 12-month SMART-ALD intervention period. Three patients were excluded from the analysis due to missing diagnostic assessment at T2. The participants showed significant improvement in the SF-36 mental health component (*p* = 0.012) and the subscales general health (*p* = 0.010), vitality (*p* < 0.001), mental health (*p* = 0.019), and GSE total scores (*p* = 0.003) from T0 to T1, but not from T1 to T2 (*ps* ⩾ 0.050). Significant improvements from T0 to T1 were also detected for satisfaction with current treatment of physical symptoms (*p* = 0.049), knowledge of balanced nutrition (*p* = 0.015), and socio-medical benefits for disability (*p* < 0.001), which increased further from T1 to T2 but without reaching statistical significance (*ps* ⩾ 0.050). Satisfaction with current treatment of psychological symptoms at T1 did not differ from T0 (*p* = 0.225), but significantly increased from T0 to T2 (*p* = 0.011). The MFIS scores decreased from T0 to T1 (*p* = 0.013), but increased again significantly from T1 to T2 (*p* = 0.013), thus returning to the T0 level.

**Table 2.** Changes in the diagnostic assessments over 12 months SMART-ALD intervention in the EG (*n* = 31).

Outcome measures	T0	T1	T2	*F*	*df*	*p*	η^2^
	*M* (SD)	*M* (SD)	*M* (SD)				
Primary outcome							
Quality of life (SF-36)							
Physical health component	37.0 (9.3)	37.2 (9.1)	34.8 (9.9)	2.378	2, 28	0.111	0.15
Mental health component	45.4 (10.0)^a^	50.4 (9.9)^b^	49.5 (10.7)^a,b^	4.863	2, 28	**0.015**	0.26
Physical functioning	49.0 (26.8)	46.4 (29.4)	45.5 (27.7)	1.182	2, 29	0.321	0.08
Role-physical	45.0 (38.5)	50.0 (33.5)	37.5 (39.3)	1.688	2,28	0.203	0.11
Bodily pain	58.6 (23.3)	61.1 (21.8)	56.8 (18.8)	0.621	2, 29	0.545	0.04
General health	45.7 (17.5)^a^	54.9 (18.6)^b^	49.6 (19.3)^a,b^	5.486	2, 29	**0.010**	0.27
Vitality	41.1 (13.3)^a^	51.3 (18.1)^b^	47.3 (15.0)^b^	12.197	2, 29	**<0.001**	0.46
Social functioning	65.3 (23.9)	72.6 (22.5)	70.2 (22.3)	2.375	2, 29	0.111	0.14
Role-emotional	60.0 (39.5)	68.9 (40.1)	63.3 (44.9)	0.617	2, 28	0.547	0.04
Mental health	63.3 (14.6)^a^	70.2 (15.8)^b^	69.3 (14.7)^b^	5.493	2, 28	**0.010**	0.28
Secondary outcome							
Self-report questionnaires							
Bladder dysfunction (ICIQ-FLUTS)	18.6 (7.3)	18.0 (5.6)	18.7 (5.8)	0.521	2, 23	0.601	0.04
Sleep quality (PSQI)	7.5 (2.8)	6.9 (3.1)	8.1 (3.0)	2.791	2, 29	0.078	0.16
Fatigue (MFIS)	35.6 (13.6)^a^	30.8 (15.7)^b^	35.8 (15.3)^a^	5.728	2, 28	**0.008**	0.29
Depression (BDI-II)	11.0 (5.8)^a^	8.5 (6.4)^b^	9.9 (7.3)^a,b^	4.398	2, 28	**0.022**	0.24
Self-efficacy (GSE)	28.1 (4.8)^a^	30.3 (4.3)^b^	29.5 (4.6)^a,b^	6.639	2, 29	**0.004**	0.31
Satisfaction with current treatment of physical symptoms (1*–10)	4.7 (2.6)^a^	5.8 (2.7)^b^	6.3 (2.5)^b^	5.334	2, 29	**0.011**	0.27
Satisfaction with current treatment of psychological symptoms (1*–10)	5.4 (3.0)^a^	6.3 (3.1)^a,b^	6.9 (2.9)^b^	4.802	2, 27	**0.016**	0.26
Knowledge of balanced nutrition (1*–10)	5.4 (2.2)^a^	6.7 (1.9)^b^	7.4 (1.4)^b^	9.566	2, 29	**<0.001**	0.40
Knowledge of socio-medical benefits for disability (1*–10)	4.8 (2.8)^a^	6.7 (2.3)^b^	7.2 (2.2)^b^	12.975	2, 25	**<0.001**	0.51
Objective measures							
Disease severity (AACS)	5.4 (2.8)	5.3 (2.4)	5.4 (2.4)	0.386	2, 29	0.683	0.03
Fitness tracker data							
Number of steps	9229.6 (5155.1)	9509.4 (5600.8)	9105.5 (5721.8)	0.272	2, 18	0.765	0.03
Light physical activity	295.6 (107.0)	280.4 (97.3)	280.9 (111.3)	2.085	2, 17	0.155	0.20
Moderate physical activity	22.1 (19.5)	21.0 (20.3)	22.6 (21.3)	0.192	2, 17	0.827	0.02
Intense physical activity	8.0 (8.3)	15.1 (18.2)	12.5 (15.5)	3.513	2, 17	0.053	0.30
Active calories	1015.0 (450.8)	1008.9 (444.1)	1008.1 (494.3)	0.022	2, 17	0.978	0.00

Statistically significant *p*-values are indicated in bold. Superscripts that differ display significant differences between time points after post hoc comparisons with Bonferroni corrections. Effect size η^2^ was interpreted according to Cohen (small effect: 0.01 ⩽ η^2^ < 0.06, medium: 0.06 ⩽ η^2^ < 0.14, large: η^2^ ⩾ 0.14).^20^

AACS, Adult Adrenoleukodystrophy Clinical Score; BDI-II, Beck Depression Inventory; EG, experimental group; GSE, General Self-Efficacy Scale; ICIQ-FLUTS, International Consultation on Incontinence Questionnaire Female Lower Urinary Tract Symptoms Module; MFIS, Modified Fatigue Impact Scale; PSQI, Pittsburgh Sleep Quality Index; SF-36, Short Form (36) Health Survey.

### Participation in individual SMART-ALD modules and effects

Table 3 shows the frequency of use of the individual counseling modules of SMART-ALD and their combination chosen by the participants of the EG (after T0), the WL-CG (after T1), and both groups together (6m IG) during the first 6-month online intervention. Sociodemographic characteristics, disease severity, QoL, and the severity of patients’ physical and mental comorbidities reported before the start of the intervention had no significant effects on the number of SMART-ALD modules selected by participants (*ps* ⩾ 0.050; Table 4). Participants who attended all four multidisciplinary counseling offers tended to be older (*p* = 0.103, η^2^ = 0.10), and reported lower physical-related QoL before intervention (*p* = 0.178, η^2^ = 0.08), but these differences of medium effect size were not statistically significant. In addition, there were significant effects of the number of SMART-ALD modules selected on changes in the SF-36 mental health component (*p* = 0.037, η^2^ = 0.14) and BDI scores (*p* = 0.015, η^2^ = 0.17) within the first 6 months of SMART-ALD participation, but without clear directional interpretability. No further group effects regarding the number of modules attended were significant (*ps* ⩾ 0.050).

**Table 3.** Frequency of participation in SMART-ALD intervention modules within the first 6 months.

SMART-ALD module selection	EG (*n* = 34)	WL-CG (*n* = 33)	6m IG (*n* = 67)
Participation frequency in individual modules			
Psychological counseling, *n* (%)	13 (38.2)	20 (60.6)	33 (49.3)
Number of sessions, *M* (SD), range	2.9 (1.4), 1–5	2.9 (2.4), 1–10	
Total time in minutes, *M* (SD), range	132.9 (56.6), 45–240	133.3 (112.3), 28–435	
Social counseling, *n* (%)	24 (70.6)	25 (75.8)	49 (73.1)
Number of sessions, *M* (SD), range	2.7 (2.6), 1–12	3.0 (2.2), 1–12	
Total time in minutes, *M* (SD), range	158.9 (94.4), 50–502	173.7 (96.2), 60–472	
Nutritional counseling, *n* (%)	25 (73.5)	26 (78.8)	51 (76.1)
Number of sessions, *M* (SD), range	1.2 (0.5), 1–3	1.1 (0.4), 1–2	
Total time in minutes, *M* (SD), range	76.2 (58.4), 35–315	74.4 (48.8), 30–245	
Fitness training, *n* (%)	19 (55.9)	19 (57.6)	38 (56.7)
Number of sessions, *M* (SD), range	12.5 (5.3), 4–22	15.8 (7.2), 3–35	
Total time in minutes, *M* (SD), range	569.3 (265.4), 160–951	643.4 (298.0), 80–1360	
Number of modules taken			
One intervention module, *n* (%)	6 (17.6)	3 (9.1)	9 (13.3)
Psychological counseling, *n* (%)	1 (2.9)	0 (0)	1 (1.5)
Social counseling, *n* (%)	0 (0)	1 (3.0)	1 (1.5)
Nutritional counseling, *n* (%)	2 (5.9)	2 (6.1)	4 (5.8)
Fitness training, *n* (%)	3 (8.8)	0 (0)	3 (4.5)
Two intervention modules, *n* (%)	8 (23.5)	9 (27.3)	17 (23.9)
Psychological counseling + nutritional counseling, *n* (%)	0 (0)	1 (3.0)	1 (1.5)
Psychological counseling + fitness training, *n* (%)	0 (0)	1 (3.0)	1 (1.5)
Social counseling + nutritional counseling, *n* (%)	5 (14.7)	4 (12.1)	9 (13.4)
Social counseling + fitness training, *n* (%)	2 (5.9)	1 (3.0)	3 (4.5)
Nutritional counseling + fitness training, *n* (%)	1 (2.9)	2 (6.1)	3 (4.5)
Three intervention modules, *n* (%)	13 (38.2)	7 (21.2)	20 (29.9)
No psychological counseling, *n* (%)	6 (17.6)	2 (6.1)	8 (11.9)
No social counseling, *n* (%)	1 (2.9)	0 (0)	1 (1.5)
No nutritional counseling, *n* (%)	1 (2.9)	1 (3.0)	2 (3.0)
No fitness training, *n* (%)	5 (14.7)	4 (12.1)	9 (13.4)
Four intervention modules, *n* (%)	5 (14.8)	12 (36.3)	17 (25.4)
No additional module used (only medical counseling), *n* (%)	2 (5.9)	2 (6.1)	4 (7.5)

6m IG, 6-month intervention group; EG, experimental group; WL-CG, waiting-list control group.

**Table 4.** Baseline characteristics and changes in outcome measures within the first 6 months of SMART-ALD intervention depending on the number of modules with participation.

Baseline characteristics and changes in outcome measures	One module (*n* = 9)	Two modules (*n* = 16)	Three modules (*n* = 21)	Four modules (*n* = 17)	*F*	*df*	*p*	η^2^
	*M* (SD)	*M* (SD)	*M* (SD)	*M* (SD)				
Pre-SMART-ALD characteristics								
Age (years)	54.7 (13.2)	56.4 (10.5)	52.7 (8.6)	60.9 (9.6)	2.153	3, 62	0.103	0.10
Education (school years)	11.1 (1.4)	10.6 (1.3)	10.8 (1.5)	11.1 (1.9)	0.333	3, 62	0.802	0.02
Disease severity (AACS)	4.8 (2.0)	5.0 (2.9)	5.8 (2.4)	5.1 (2.4)	0.510	3, 61	0.677	0.03
Physical health component (SF-36)	40.1 (9.6)	37.6 (8.5)	34.9 (10.6)	31.6 (11.3)	1.693	3, 62	0.178	0.08
Mental health component (SF-36)	46.4 (7.9)	47.3 (10.6)	42.9 (9.7)	48.2 (10.6)	1.033	3, 62	0.385	0.05
Bladder dysfunction (ICIQ-FLUTS)	12.6 (7.4)	13.6 (7.6)	18.8 (8.5)	13.1 (6.6)	2.408	3, 59	0.077	0.11
Sleep quality (PSQI)	7.8 (2.7)	8.9 (3.2)	8.5 (3.2)	7.4 (3.2)	0.708	3, 61	0.551	0.04
Fatigue (MFIS)	35.7 (16.5)	32.2 (15.5)	36.0 (16.3)	32.4 (17.9)	0.241	3, 61	0.868	0.01
Depression (BDI-II)	10.4 (7.0)	10.1 (8.3)	13.4 (8.6)	10.8 (7.0)	0.669	3, 62	0.574	0.03
Self-efficacy (GSE)	30.4 (3.8)	28.4 (5.7)	27.0 (5.1)	30.5 (5.4)	1.755	3, 61	0.166	0.08
Changes within 6-month SMART-ALD								
Quality of life (SF-36)								
Δ Physical health component	1.0 (4.8)	0.3 (4.8)	−2.2 (7.6)	2.8 (6.9)	1.902	3, 60	0.139	0.09
Δ Mental health component	5.5 (9.6)^a,b^	−2.2 (7.1)^a^	6.3 (8.8)^b^	1.6 (10.3)^a,b^	3.015	3, 60	**0.037**	0.14
Δ Physical functioning	1.1 (12.4)	−2.5 (7.1)	−2.2 (13.1)	2.1 (12.9)	0.631	3, 62	0.589	0.03
Δ Role-physical	5.6 (24.3)	−10.9 (31.6)	−2.5 (45.8)	11.8 (39.6)	1.064	3, 61	0.372	0.05
Δ Bodily pain	1.2 (16.1)	4.5 (20.2)	−4.7 (15.3)	5.6 (16.0)	1.439	3, 62	0.241	0.07
Δ General health	11.7 (17.2)	2.4 (14.5)	7.8 (14.7)	7.6 (14.5)	0.830	3, 62	0.483	0.04
Δ Vitality	8.9 (10.8)	0.0 (9.7)	8.5 (15.7)	6.2 (8.6)	1.827	3, 61	0.152	0.09
Δ Social functioning	13.9 (16.3)	−3.9 (16.3)	12.5 (22.7)	5.1 (17.1)	2.778	3, 62	0.049	0.12
Δ Role-emotional	18.5 (50.3)	−12.5 (40.1)	10.0 (47.3)	−3.9 (49.8)	1.174	3, 61	0.328	0.06
Δ Mental health	3.1 (12.6)	−2.1 (10.1)	7.6 (12.1)	6.4 (12.6)	2.220	3, 60	0.096	0.11
Self-report questionnaires								
Δ Bladder dysfunction (ICIQ-FLUTS)	1.9 (4.1)	0.49 (3.2)	−1.6 (4.5)	−0.6 (4.2)	1.643	3, 51	0.192	0.09
Δ Sleep quality (PSQI)	−0.3 (2.0)	−0.5 (2.5)	−0.7 (3.3)	−0.3 (2.0)	0.079	3, 60	0.971	0.00
Δ Fatigue (MFIS)	−9.9 (9.7)	−1.4 (9.0)	−3.0 (11.8)	0.1 (10.5)	1.876	3, 60	0.144	0.09
Δ Depression (BDI-II)	−2.6 (4.1)^a,b^	3.1 (4.9)^a^	−1.3 (6.1)^a,b^	−1.7 (3.9)^b^	3.804	3, 60	**0.015**	0.17
Δ Self-efficacy (GSE)	−0.7 (3.1)	1.5 (4.0)	2.1 (4.2)	0.4 (2.6)	1.446	3, 60	0.239	0.07
Δ Satisfaction with current treatment of physical symptoms	0.6 (1.3)	1.4 (3.0)	1.5 (2.6)	1.6 (2.1)	2.556	3, 61	0.738	0.02
Δ Satisfaction with current treatment of psychological symptoms	1.2 (2.5)	1.0 (2.3)	2.0 (3.2)	1.5 (2.5)	0.409	3, 62	0.747	0.02
Δ Knowledge of disease-specific nutrition	−0.3 (1.1)	1.4 (2.2)	1.1 (1.8)	1.2 (2.0)	1.812	3, 61	0.155	0.09
Δ Knowledge of socio-medical benefits for disability	0.7 (1.4)	2.0 (2.9)	2.0 (1.9)	1.3 (1.7)	0.927	3, 57	0.434	0.05
Fitness tracker data								
Δ Number of steps	1024.8 (1891.3)	412.5 (2113.6)	801.9 (2200.7)	98.2 (1350.2)	0.442	3, 46	0.724	0.03
Δ Light physical activity	−10.2 (15.7)	−12.1 (47.5)	26.7 (48.6)	−8.5 (25.8)	2.809	3, 45	0.051	0.17
Δ Moderate physical activity	−3.6 (9.7)	1.4 (7.3)	0.5 (6.9)	1.2 (7.7)	0.593	3, 45	0.623	0.04
Δ Intense physical activity	4.4 (4.6)	6.5 (14.0)	2.3 (7.5)	1.2 (4.9)	0.845	3, 45	0.477	0.06
Δ Active calories	−20.6 (54.1)	5.2 (169.5)	109.9 (176.2)	−1.4 (163.2)	1.618	3, 45	0.200	0.10
Evaluation rating (T2)								
Total score	2.6 (1.0)	2.4 (1.1)	2.6 (0.7)	2.7 (0.9)	0.372	3, 58	0.774	0.02

Δ = Difference score of 6-month Post-SMART-ALD minus Pre-SMART-ALD; negative change values are associated with a decrease in the questionnaire/clinical score or activity measured by the fitness trackers, positive values with an increase in the scores in the respective questionnaire or physical activity measured by fitness trackers. Statistically significant *p*-values are indicated in bold. Superscripts that differ display significant differences between groups after post hoc comparisons with Bonferroni corrections. Effect size η^2^ was interpreted according to Cohen (small effect: 0.01 ⩽ η^2^ < 0.06, medium: 0.06 ⩽ η^2^ < 0.14, large: η^2^ ⩾ 0.14).^20^

AACS, Adult Adrenoleukodystrophy Clinical Score; BDI-II, Beck Depression Inventory; GSE, General Self-Efficacy Scale; ICIQ-FLUTS, International Consultation on Incontinence Questionnaire Female Lower Urinary Tract Symptoms Module; MFIS, Modified Fatigue Impact Scale; PSQI, Pittsburgh Sleep Quality Index; SF-36, Short Form (36) Health Survey.

### Feasibility and acceptance

Of the 97 self-registrants who were interested in this study, *n* = 5 (5.2%) were excluded from participation due to a lack of technical equipment for video consultations. One participant of the WL-CG dropped out after the waiting period, and two participants of the EG stopped SMART-ALD participation after 6 months. The adherence rate for SMART-ALD was thus 95.6%. Results of participants’ evaluation rating of the SMART-ALD intervention are displayed in Table 5. The evaluation of the individual seven items and the total score of the evaluation did not differ between EG and WL-CG (*p* = 0.266–0.980, *d* = 0.02–0.30). Patient’s total time of participation in SMART-ALD modules positively correlated with age (*r* = 0.25, *p* = 0.046) and scores on item #2 (*r* = 0.40, *p* = 0.001), #3 (*r* = 0.48, *p* < 0.001), #4 (*r* = 0.36, *p* = 0.004), and the total score (*r* = 0.39, *p* = 0.002) of the evaluation questionnaire. Age (*r* = −0.34, *p* = 0.007) and AACS at baseline (*r* = −0.27, *p* = 0.034) were negatively associated with scores on item #7 at evaluation. Regarding person-related characteristics, the SF-36 mental health component assessed before intervention was positively correlated with evaluation scores on item #1 (*r* = 0.41, *p* = 0.001), #4 (*r* = 0.38, *p* = 0.005), #6 (*r* = 0.31, *p* = 0.014), #7 (*r* = 0.28, *p* = 0.028), and the total score (*r* = 0.33, *p* = 0.008). In contrast, MFIS and BDI-II scores were negatively correlated with scores on item #1 (*r* = −0.36, *p* = 0.004 and *r* = −0.35, *p* = 0.005), #2 (*r* = −0.30, *p* = 0.017 and *r* = −0.28, *p* = 0.026), #3 (*r* = −0.25, *p* = 0.044 and *r* = −0.27, *p* = 0.034), #4 (*r* = −0.38, *p* = 0.002 and *r* = −0.38, *p* = 0.002), and the total evaluation score (*r* = −0.33, *p* = 0.008 and *r* = −0.37, *p* = 0.003). A negative link was also found between BDI-II scores before intervention and scores on item #7 (*r* = −0.31, *p* = 0.015), whereas the GSE score before intervention was positively associated with scores on item #7 (*r* = 0.27, *p* = 0.032). No other significant correlations were observed.

**Table 5.** Results of the participants’ evaluation of the SMART-ALD intervention at T2.

Evaluation rating items	Do not agree at all (0)	Hardly agree (1)	Partially agree (2)	Largely agree (3)	Totally agree (4)	Total score (0–4)
	*n* (%)	*n* (%)	*n* (%)	*n* (%)	*n* (%)	*M* (SD)
1. SMART-ALD represents more intensive care for women with AMN/X-ALD, which I consider important as an addition to regular follow-up checks at a leukodystrophy outpatient clinic.	0 (0.0)	5 (7.5)	3 (4.5)	19 (28.4)	36 (53.7)	3.4 (0.9)
2. My physical well-being has improved by participating in SMART-ALD.	12 (17.9)	7 (10.4)	21 (31.3)	17 (25.4)	6 (9.0)	2.0 (1.2)
3. My mental well-being has improved by participating in SMART-ALD.	14 (20.9)	9 (13.4)	16 (23.9)	18 (26.9)	6 (9.0)	1.9 (1.3)
4. My quality of life has improved by participating in SMART-ALD.	11 (16.4)	8 (11.9)	22 (32.8)	17 (25.4)	5 (7.5)	2.0 (1.2)
5. By participating in SMART-ALD, I know what I can do myself to prevent the disease from progressing.	5 (7.5)	4 (6.0)	18 (26.9)	18 (26.9)	18 (26.9)	2.6 (1.2)
6. My expectations regarding SMART-ALD have been fulfilled.	1 (1.5)	4 (6.0)	16 (23.9)	27 (40.3)	15 (22.4)	2.8 (0.9)
7. Participation in SMART-ALD via video (without face-to-face meetings) was unproblematic.	2 (3.0)	3 (4.5)	6 (9.0)	17 (25.4)	35 (52.2)	3.3 (1.0)

The analysis is based on *n* = 63 data sets. Three patients in the experimental group and one patient in the control group did not complete the questionnaire in time.

Regarding human resources, the implementation of 12-month SMART-ALD for the current sample required an average of 7 working hours per week (h/w) for medical staff, 3 h/w for psychology, 2 h/w for nutritional counseling, 4 h/w for social work, and 20 h/w for sports therapists. Most patients required closer supervision at the beginning of the SMART-ALD program. However, the frequency of sessions across all disciplines, and thus the use of study personnel time and resources, generally decreased once the medical treatment recommendations were implemented by the local physicians and the lifestyle changes were carried out independently by the patients and integrated into their daily routines.

## Discussion

Improving QoL may be the most important treatment goal for people living with chronic, progressive, and incurable RDs.^21^ This study showed that a multidisciplinary, easily accessible, and individually tailored lifestyle intervention for symptomatic women with X-ALD leads to improvements in self-reported QoL, well-being, self-efficacy, treatment satisfaction, and physical activity after only 6 months of participation. This is consistent with findings on the efficacy of telemedicine in conditions with similar symptoms, for example, multiple sclerosis (MS).^22,23^

The largest effects of 6-month SMART-ALD intervention were detected on self-reported vitality measured by SF-36. The EG reported medium-to-large-sized improvements on the SF-36 mental health component and the subscales general health and social functioning after 6-month SMART-ALD. Slight, albeit non-significant improvements in these domains were also reported in the WL-CG so that the rmANOVA did not reveal any significant interaction effects. However, improvements in the WL-CG can be attributed to the expectation of more intensive care after the waiting period. Moreover, the perception of the study team’s awareness of and interest in their state of health at the diagnostic appointments (T0, T1) may also have led to positive effects on well-being.

Regarding secondary outcomes, 6-month SMART-ALD participation led to a significant increase in knowledge of balanced nutrition and socio-medical benefits for disability in the EG versus WL-CG. Additionally, improvements of fatigue, depression, self-efficacy, and satisfaction with current physical and psychological treatment were found over time in both groups, with medium-to-large-sized time effects being statistically significant only in the EG. Here again, the pure intervention effects may have been attenuated by waiting-list effects.

No intervention effects were found on self-reported burden of bladder dysfunction and frequently associated sleep disturbances. Here, patients with these complaints often were referred to a specialist discipline (e.g., continence center). Not all patients were able to implement this recommendation within the 6-month SMART-ALD participation period, for example, due to waiting times at those specialized centers in Germany.^24^ Apart from the increase in daily intensive physical activity in the EG versus WL-CG at T1, there was no significant improvement in other objective measures. This can be attributed to seasonal effects on patients’ physical activity that affected both groups. The T0 measurement took place in summer (July/August 2022), while the T1 measurements were conducted 6 months later in winter, when physical activity is naturally reduced due to shorter days and poorer weather conditions (snow, black ice), especially in patients with an increased risk of falling due to balance and gait disorders.^25,26^ Accordingly, the current results suggest positive, albeit small effects of SMART-ALD on physical activity, as the EG maintained stable activity levels even in winter, while the WL-CG showed a slight decrease in the number of daily steps, minutes of light and moderate activity, and active calories at T1.

The long-term analysis within the EG showed that the improvements in QoL and well-being achieved after 6 months were maintained after 12-months SMART-ALD participation. Interestingly, fatigue increased again between 6 and 12 months returning to the pre-intervention level. These fluctuations could be interpreted as seasonal. Studies in the MS field have already shown that fatigue in patients increases at higher outside temperatures.^27^

Regarding the impact of person-related baseline characteristics on the selection of SMART-ALD modules, older and more physically impaired participants were more likely to complete the full SMART-ALD program, likely due to greater myeloneuropathy, increased distress, and more available time from age- or disease-related occupational disability.^7^ No effect of the number of modules selected was found on the changes achieved after 6-month SMART-ALD, suggesting that tailoring the modules to individual needs is more important than quantity. These findings highlight that intensive additional online programs like SMART-ALD are especially valuable for patients with severe disease burden and limited access to local care due to age or immobility. Besides reviewing eligibility criteria, tailoring the program to individual needs also contributes to cost-effectiveness by directing resources to the areas of patients’ lives with the greatest gaps in care, rather than overwhelming all patients with multiple treatments regardless of their needs.

The feasibility and compliance in this study were very high, with only 5% of patients not being included due to a lack of technical requirements, and another 5% dropping out after 6-month active participation. Based on the SMART-ALD evaluation rating, the majority of participants largely agreed that SMART-ALD provided a beneficial addition to regular on-site follow-up at a CoE (82.1%), they were taught strategies for self-efficacy against disease progression (53.8%), their expectations of the intervention were met (62.7%), and online-only participation was not a barrier (77.6%).

Nevertheless, a substantial proportion stated that participation in SMART-ALD had not improved their physical (28.3%) and mental (34.4%) well-being and QoL (28.3%). Besides a higher total participation time in SMART-ALD modules being linked to greater improvement in well-being, differences in the benefits from SMART-ALD also resulted from patients’ baseline characteristics. In particular, high levels of depression and fatigue in patients at baseline were associated with low improvement in well-being and QoL from SMART-ALD participation. Depression and low self-efficacy were also associated with a poor willingness to use online-only treatment offers.

Strengths of this study include the prospective, longitudinal, randomized-controlled design, and the multidimensional assessment of subjectively reported and objectively measured outcomes in a relatively large sample given a RD. However, the present sample was too small to detect statistically significant intervention effects of small-to-medium size. Other limitations include the lack of recording of objective myeloneuropathy outcomes such as strength or gait measures, which was not possible with remote assessments. Both groups reported a decline in QoL regarding physical functioning of medium effect size within the first 6 months. This illustrates the disease’s progressive nature,^4–6^ which cannot be halted by the treatment currently available. However, differences in physical decline between treated and untreated groups might become apparent with larger study samples and by applying sensitive objective measures like instrumented gait analysis or postural sway.^28^

Future randomized-controlled clinical trials should plan longer interventions with more frequent on-site follow-ups at the CoE to test objectively measured effects of online programs on disease progression. Furthermore, the cost-effectiveness of additional web-based services to intensify the care for people with RDs compared to existing care still remains to be evaluated in detail in the long term.

## Conclusion

This study provides promising initial results demonstrating that a web-based, disease-specific, multidisciplinary lifestyle intervention provided by a CoE has the potential to improve the QoL of symptomatic women with X-ALD, even after only 6 months of participation. This study was conducted in symptomatic women with X-ALD who had been identified as a patient group particularly susceptible to misdiagnosis and undertreatment out of a CoE. However, inappropriate care has also been identified in other RDs affecting both sexes for similar reasons and thus, we believe our findings are highly relevant to improving care for this patient group in general. As the diagnoses of genetic RDs continuously improve and life expectancy increases,^29,30^ it is crucial to leverage and expand the use of telemedicine to close care gaps and provide more intensive multidisciplinary support for people with RDs who live far from their CoE.

## Supplemental Material

sj-doc-1-tan-10.1177_17562864251376109 – Supplemental material for Improving quality of life in rare diseases using disease-specific, multidisciplinary online interventions on the example of rare X-linked adrenoleukodystrophy: a randomized-controlled trial Supplemental material, sj-doc-1-tan-10.1177_17562864251376109 for Improving quality of life in rare diseases using disease-specific, multidisciplinary online interventions on the example of rare X-linked adrenoleukodystrophy: a randomized-controlled trial by Lisa Schäfer, Astrid Unterlauft, Brit Froebrich-Andreß, Carolin Wollny, Marie Rößler, Ronja Fischer, Carla Bähr, Julia Lier, Daniel T. Wasmus, Christa-Caroline Bergner and Wolfgang Köhler in Therapeutic Advances in Neurological Disorders

## Declarations

**Ethics approval and consent to participant:** This study was approved by the Ethical Committee at the Medical Faculty, Leipzig University, Germany on January 19, 2021 (592/20-ek). Patients’ written informed consent was obtained before study enrollment.

**Consent for publication:** Not applicable.

**Availability of data and materials:** The data that support the findings of this study are available from the corresponding author upon reasonable request.
